# Trimethoprim-sulfamethoxazole induced aseptic meningitis case report

**DOI:** 10.1097/MD.0000000000032475

**Published:** 2023-01-06

**Authors:** Giulia Pata, Marco Montagna, Emanuele Bosi, Alberto Davalli, Patrizia Rovere Querini

**Affiliations:** a Vita-Salute San Raffaele University, Milan, Italy; b Diabetes and Endocrinology Unit, Department of Internal Medicine, IRCCS San Raffaele Scientific Institute, Milan, Italy.

**Keywords:** aseptic meningitis, case report, drug-induced aseptic meningitis (DIAM), trimethoprim-sulfamethoxazole

## Abstract

Drug-induced aseptic meningitis (DIAM) is an uncommon meningitis and trimethoprim with or without sulfamethoxazole is the most involved antibiotic. Although DIAM is easily treated with the discontinuation of the causative drug, the diagnosis is a big challenge for physicians, as it remains a diagnosis of exclusion. Here, we present a case report of trimethoprim-sulfamethoxazole induced aseptic meningitis in a woman with acute osteomyelitis.

**Patient concerns:**

A 52-year-old woman was admitted to the hospital for septic shock and acute osteomyelitis of the right homerus. She was started on antibiotic therapy with oxacillin and daptomycin, then oxacillin was replaced with cotrimoxazole, due to its excellent tissue penetration, including bone tissue. During cotrimoxazole therapy, the patient developed a fluent aphasia with ideomotor apraxia and muscle hypertonus.

**Diagnosis and interventions:**

Having excluded infectious, epileptic and vascular causes of the acute neurologic syndrome of our patient, given the improvement and full recovery after discontinuation of cotrimoxazole, we hypothesized a DIAM.

**Outcomes:**

After discontinuation of cotrimoxazole, in 48 hours the patient had a full recovery.

**Lessons:**

Although DIAM can be easily managed with the withdrawal of the causative drug, it can be difficult to recognize if it is not included in the differential diagnosis. An antimicrobial stewardship program with a strict monitoring of patients by infectious disease specialists is essential, not only to optimize the appropriate use of antimicrobials, but also to improve patient outcomes and reduce the likelihood of adverse events.

## 1. Introduction

Trimethoprim with or without sulfamethoxazole is the most common antibiotic implicated in drug-induced aseptic meningitis (DIAM).^[1]^ The exact mechanism causing trimethoprim-sulfamethoxazole (TMP-SMX) aseptic meningitis is unclear, but predisposing factors include autoimmune diseases, preexisting diabetes mellitus and immunosuppression.^[2–4]^ The most common symptoms are fever, headache, meningism, and mental status changes. Symptoms remitted with the withdrawal of TMP-SMX, over 48 to 72 hours.^[3,5]^

Here, we present a case report of TMP-SMX induced aseptic meningitis in a woman with acute osteomyelitis.

## 2. Case presentation

A 52-year-old Italian woman with a past medical history of poorly controlled diabetes mellitus presented to the Emergency Department of San Raffaele Hospital in Milan for hyperpyrexia with confusion and delirium.

At the arrival she was febrile, with raised inflammatory indexes (C-reactive protein: 318 mg/L) and marked hyperglycemia (blood glucose: 705 mg/dL). Blood cultures were performed, with isolation of methicillin-susceptible *Staphylococcus aureus* (MSSA).

Due to the onset of septic shock requiring vasoactive medications, she was admitted to the intensive care unit (ICU). During her ICU stay she was started on antibiotic therapy with oxacillin and daptomycin. Hyperglycemia was treated with insulin therapy, initially intravenous and then subcutaneous.

A cerebrospinal fluid (CSF) analysis revealed a moderate hyperglycorrachia, with negative cultures and negative viral tests. In consideration of recent dental treatments and of the finding of MSSA in the blood cultures, a transesophageal echocardiography was performed, ruling out an endocarditis. A total body computed tomography (CT) scan excluded infectious foci.

After 5 days the patient was moved to our Internal Medicine Unit. There, she continued the antibiotic therapy started in ICU. The patient complained of a pain in her right arm, which appeared to be swollen, so a venous doppler ultrasound was performed, which revealed a deep vein thrombosis of right axillary and brachial veins. Accordingly, she started anticoagulation with enoxaparin.

After 10 days, blood cultures were still positive for MSSA, so the patient repeated a total body CT scan, which described multiple abscesses in various muscles and soft tissues, the biggest at the level of right humeral head and subscapularis muscle. The exam also revealed lung parenchymal thickenings, some with cavitated appearance, compatible with additional infectious foci. Endocarditis was again ruled out after a transesophageal echocardiography. During her stay, the patient has been frequently evaluated by infectious disease specialists, who, considering the susceptibility of the microorganism to the ongoing antibiotics, did not suggest any correction of the therapy. Indeed, blood culture negativity was obtained in 2 weeks.

To evaluate the possibility of surgical orthopedic treatment of the abscesses, a magnetic resonance imaging of the right shoulder was performed. The exam was suggestive for acute osteomyelitis of the right humerus. However, orthopedic surgeons did not give a surgical indication, so the patient continued the antibiotic therapy. Two weeks later, after a joint discussion with infectious disease specialists, the therapy with oxacillin was replaced with cotrimoxazole, due to its excellent tissue penetration, including bone tissue.^[2]^

After 10 days of cotrimoxazole therapy, the patient developed a high fever (with peaks of 39.5°C), headache, nausea, and vomiting.

A total body CT was therefore repeated to reassess her primary infection, revealing a dimensional reduction of the soft tissues abscesses and of the lung parenchymal thickenings, and a stability of the humeral involvement.

After a few days the patient appeared aphasic and she was not able to answer simple questions properly. An urgent neurological visit was performed and the neurologist confirmed the presence of a fluent aphasia, accompanied by an ideomotor apraxia and muscle hypertonus.

The neurologist asked for an urgent brain CT scan, which excluded acute ischemic events, confirmed by a brain magnetic resonance imaging, and an electroencephalogram, which did not detect any epileptic feature.

After consulting infectious disease specialists, cotrimoxazole was discontinued and oxacillin was restarted.

Empiric antiviral therapy with intravenous aciclovir and anticomitial therapy with intravenous levetiracetam was started, based on neurological indications.

In patients with suspected meningitis, it is strongly recommended to determine CSF leukocyte count, protein and glucose concentration and to perform CSF culture and Gram stain, in order to differentiate between bacterial meningitis and other causes.^[6]^

However, due to ongoing anticoagulation therapy, lumbar puncture was performed after 24 hours of discontinuation of enoxaparin.

The CSF analysis finally revealed an elevated CSF protein level (151 mg/dL), with negative cultures and viral tests.

In 48 hours the patient had a full recovery.

Having excluded infectious, epileptic, and vascular causes of the acute neurologic syndrome of our patient, given the improvement and full recovery after discontinuation of cotrimoxazole, we hypothesized a DIAM.

## 3. Discussion

Trimethoprim with or without sulfamethoxazole is the most common antibiotic implicated in DIAM. The TMP-SMX aseptic meningitis was first described in 1983 by Kremer at al.^[1]^

In a review of the literature from 1990 to 2019 performed by Corsini Campioli at al,^[3]^ a total of 42 cases of TMP-SMX aseptic meningitis were collected.

Most patients were female (59.2%). The most common symptoms reported were fever, headache, meningism and mental status changes. The CSF analysis typically demonstrated a neutrophilic pleocytosis and elevated CSF protein level. Symptoms remitted with the withdrawal of TMP-SMX, over 48 to 72 hours.^[3,5]^

In another review done by Moris et al^[7]^ TMP-SMX was the antibiotic most associated with DIAM (62.3%), with a range of latency of 10 minutes to 3 months after exposure. In addition to the symptoms listed above, focal neurologic deficits were described in 7% of the antibiotic-induced aseptic meningitis (in this group we recognize the acute aphasia of our patient).

Our patient additionally presented generalized muscular hypertonia: similar cases were already reported, as the one described by Capra C. et al.^[8]^

Predisposing factors of TMP-SMX induced aseptic meningitis include autoimmune diseases, preexisting diabetes mellitus, and immunosuppression (resulting from human immunodeficiency virus infection, chemotherapy or organ transplantation).^[2–4]^

Our patient had 2 predisposing factors: diabetes mellitus and autoimmune disease.

Diagnosis of diabetes mellitus was established in 2010, at the age of 40. At the time of the admission her glycated hemoglobin was 126 mmol/mol. We found the presence of autoantibody against glutamic acid decarboxylase, that, together with the young age at the diagnosis, the absence of metabolic syndrome and non-insulin requiring at onset, is very suggestive of latent autoimmune diabetes of adults.^[9]^

The exact mechanism causing TMP-SMX aseptic meningitis is unclear. Immune complex deposition, type III or IV hypersensitivity, direct drug toxicity and induction of autoantibodies have all been hypothesized.^[3,4,8,10–12]^ Interleukin-6 seems to be an important mediator, with elevated levels in serum and CSF in these patients.^[13]^

## 4. Conclusion

DIAM is an uncommon meningitis and TMP-SMX is the most involved antibiotic. Although this type of meningitis is easily treated with the discontinuation of the causative drug, the diagnosis remains a big challenge for physicians, as it remains a diagnosis of exclusion.^[1,14,15]^

This case report underlines how an antimicrobial stewardship program with a strict monitoring of patients by infectious disease specialists is essential, not only to optimize the appropriate use of antimicrobials decreasing the emergence and spread of multidrug-resistant infections, but also to improve patient outcomes and reduce the likelihood of adverse events.^[16–18]^

## Author contributions

**Conceptualization:** Giulia Pata, Marco Montagna, Emanuele Bosi, Alberto Davalli, Patrizia Rovere Querini.

**Data curation:** Giulia Pata, Alberto Davalli.

**Writing – original draft:** Giulia Pata.

**Writing – review & editing:** Marco Montagna, Emanuele Bosi, Patrizia Rovere Querini.
